# Orthoform—A Local Anesthetic for Open Wounds, Burns, Ulcers, Etc.

**Published:** 1898-10

**Authors:** A. Einhorn, R. Heinz

**Affiliations:** Munich; Munich


### SELECTIONS.

### Orthoform—A Local Anesthetic for Open Wounds, Burns, Ulcers, Etc.

BY DR. A. EINHORN AND DR. R. HEINZ, MUNICH.

A local anesthetic for the relief of pain in open wounds, ulcers, burns, fissures, excoriations, etc., must possess the following properties: In the first place, it must be absolutely non-toxic, and secondly, it must be difficult, i. e., *slow of absorption.* The cocaine salts do not fill these requirements. On account of their toxicity, they may be used in but very limited quantities, and as they are readily soluble they are quickly absorbed, so that their anesthetic effect is lost after 15 minutes to one hour.

We have succeeded, after researches extending over a number of years, in finding a substance which is none-toxic, and produces complete local anesthesia for a prolonged length of time, the latter advantage being due to the fact that the remedy is but slightly soluble (like iodoform) and therefore remains at the place of application a more or less prolonged period of time, continuing its anesthetic influence till it is finally completely absorbed. This body, which was discovered after a searching study of the chemical constitution of cocaine, has been called "Orthoform." It is a white, tasteless and odorless powder of low specific gravity. It dissolves slowly in water and only to a slight degree. This very quality distinguishes it from all other anesthetics known. Just enough of the substance is dissolved and absorbed *in loco* to produce the desired local anesthesia. Its absorption occurs so slowly that the anesthetic effect lasts for hours and even days.

Orthoform forms, with muriatic acid, a salt which can be crystallized, and which is very readily soluble in water, its solution giving an acid reaction. The muriate of orthoform anesthetizes as well as the free base itself, but on account of the acid reaction of its solutions, the latter cannot be generally used.

They may not be applied to sensitive mucous membranes (especially of the eye), nor to the general tissues of the body, which give an alkaline reaction and are exquisitely sensitive to acids; the muriate may not be used, therefore, for hypodermic injections.

The anesthetic effect of orthoform on mucous membranes or on the nerve-ends of the skin when exposed by wounds, abrasions, burns, etc., can be readily demonstrated. When placed on the tongue, at first nothing is felt on account of its slow solubility; in a few minutes a feeling of numbness sets in which gradually increases till complete analgesia is developed at the point of contact with the powder. When applied to the eye of a rabbit a slight reddening of the conjunctiva is soon noticeable; after a few minutes, perfect anesthesia is present at all points at which the orthoform has been in direct contact with the conjunctiva; at all other points no such effect is noticeable—from which it follows that in order to obtain perfect anesthesia the remedy must be applied *equally* to all points. For this reason it is best to apply orthoform as a fine powder, or, as on wounds, ulcers, etc., embodied in an ointment.

The first very instructive experience with orthoform on man was made in a case of THIERSCH'S grafting. The patient (it was a case of lupus) had previously had four graftings made, and had each time complained for hours of very intense pain in the places from which the skin for the grafting had been taken. In the present instance, a 10 percent orthoform ointment was applied during the anesthesia to the wounded surface on the left side of the chest. Upon awakening, the patient was surprised to find himself perfectly free from pain. Two days later the dressings were changed, and the wound and surrounding skin were found free from all irritation, and there was besides very little secretion (areas of skin from which grafts have been taken are apt to show considerable soaking); the renewal of the epidermis occurred subsequently in a perfectly normal manner. The case of THIERSCH'S grafting, which was followed by similar instances with equal success, illustrates the action of orthoform very well. Orthoform is active wherever it is in immediate contact with exposed nerve-ends. It is inert on the other hand when separated from the nerve-ends by unbroken skin or a thick mucous membrane. It is useless, therefore, to apply it to a wound closed by suture, as it can not penetrate the epidermis. Thus also in cases where there is no loss of epidermis, as in burns of the first degree, it develops no analgetic properties. Its action in burns of the third degree, on the contrary, is astonishing. We have already applied it in powder form or as an ointment in a number of cases of severe burns, in which the most violent pains were quickly and permanently relieved. In these cases its tendency to keep the wound dry was striking, so that the latter healed rapidly—a great deal more rapidly than ordinarily. A number of cases in which for the sake of comparison orthoform was applied to one burnt hand and a bland borated ointment to the other were very instructive. The pains promptly disappeared in the hand dressed with the former, and continued unabated in the other. The next day the dressings were reversed, and the same result was obtained "mutatis mutandis."

The same analgetic effect was obtained upon the application of orthoform to painful ulcers. A patient with an ulcerated cancer of the face had suffered for months from violent pains, and was able to keep for the first time a whole night free from pain upon the application of orthoform. About 50 grammes of the remedy was applied in the course of a week to the ulcerated surface. This would have been impossible but for its absolute freedom from toxic properties, a fact which had been demonstrated also by experiments upon animals. Rabbits to which 2.0 to 4.0 grammes (30 to 60 grains) a day had been fed for some days, and dogs to which had been given 3 0 to 6.0 grammes (45 to 90 grains) by mouth and by hypodermic injections developed absolutely no symptoms of poisoning, remaining bright and lively and showing upon post-mortem examination absolutely no evidence of any morbid changes in any of the viscera. The case of cancer of the face, cited above, illustrates, however, even better than these experiments the total absence of toxic qualities in the drug.

Orthoform possesses another valuable property which surgeons will appreciate; it is a good antiseptic. It prevents decomposition and fermentation and stops suppuration where it is present. On this account and on account of its desiccating properties referred to above, orthoform must be considered an excellent dressing for wounds. When applied as such to ulcers of the leg, for instance, it proved very satisfactory, especially in those cases in which violent pains had been present, and were relieved by the application of orthoform.

Orthoform was also of excellent service in a number of cases of injury. In one case in which the thumb had been cut off by machinery, the pain was relieved in a few minutes by a dressing of orthoform. The pain following the extraction of a number of molar teeth disappeared quickly upon the free application of the remedy in powder form to the gums.

Besides its value in cases of extensive injury, burns and ulcers, orthoform will be found useful in a number of minor lesions, such as fissures of the lips, breasts or anus, excoriations, painful wet eczema, ulcers of the tongue, lips, etc., etc.

Laryngeal ulcerations form another grateful field for the use of orthoform. A number of patients suffering from severe pains had been treated with cocaine, which gave them but short lived-relief. After an hour the pains would return, unabated in their severity. Deglutition was especially painful in these cases, so that food could not be taken in sufficient quantities, and the general nutrition of the patients was seriously impaired. After the insufflation of orthoform analgesia was obtained for twenty-four hours, the patients took food without pain, and as a result their general condition and weight improved most satisfactorily.

*A priori* it seemed probable that orthoform could be used with success to relieve pain in the gastro-intestinal tract; and, in fact, wherever it comes into contact with exposed nerve-ends, as for instance, in round ulcer of the stomach or in ulcerated cancer of the stomach, it acts as an analgetic. On the other hand, little or nothing can be expected from it for the relief of the painful sensations accompanying catarrh and dilatation of the stomach, etc.

Whereas, for reasons stated above, orthoform itself must be considered preferable for all external applications, the readily soluble muriate of orthoform is equally useful in internal medication. Thus in the treatment of gastric ulcer and cancer of the stomach very good results were obtained from the use of the muriate of orthoform. The experiments made with the internal administration of orthoform will be published shortly by the Staff of the Royal Munich Medical Polyclinic.

There can be no doubt that the remedy will be found useful in many other fields of medicine. Wherever it is brought into immediate contact with irritated nerve-ends the latter are paralyzed and cease to pain. Further investigations are required to show whether orthoform or its muriate can be used to anesthetize the mucous membranes of the mouth, nose and throat. As to the dosage of orthoform it may be remarked that its absolute freedom from toxic properties warrants its application *ad libitum* to the most extensive wounds or ulcers. Internally thus far 0.5 to 1.0 grammes (gr. VII to gr. XV) of orthoform or its muriate have been given several times a day.

Orthoform is manufactured as a fine white powder. It is prepared by the Farbwerke vorm Meister, Lucius & Bruning, in Hoechst-on-the-Main. The powder is always ready for use; its antiseptic qualities obviate the necessity of sterilizing it before use. It does not deteriorate, is not hygroscopic, and can be dispensed according to the ordinary methods of administration.—*Munchener Med. Wochenschrift, Aug.*, 1897.

